# Association of Parental Incarceration With Psychiatric and Functional Outcomes of Young Adults

**DOI:** 10.1001/jamanetworkopen.2019.10005

**Published:** 2019-08-23

**Authors:** Elizabeth J. Gifford, Lindsey Eldred Kozecke, Megan Golonka, Sherika N. Hill, E. Jane Costello, Lilly Shanahan, William E. Copeland

**Affiliations:** 1 Center for Child and Family Policy, Duke University, Durham, North Carolina; 2 Children’s Health and Discovery Initiative, Duke University, Durham, North Carolina; 3 Department of Psychiatry and Behavioral Sciences, Duke University Medical Center, Durham, North Carolina; 4 The Jacobs Center for Productive Youth Development, Department of Psychology, University of Zurich, Zurich, Switzerland; 5 Vermont Center for Children, Youth, and Families, Department of Psychiatry, University of Vermont, Burlington

## Abstract

**Importance:**

In 2016, an estimated 8% of US children younger than 18 years had experienced the incarceration of a parent, and rates were substantially higher among children from racial and ethnic minority backgrounds and disadvantaged groups. Little is known about whether parental incarceration during childhood is associated with adult psychiatric problems and functional outcomes.

**Objective:**

To examine whether parental incarceration is associated with increased levels of psychiatric diagnosis and poor outcomes in health, legal, financial, and social domains in adulthood.

**Design, Setting, and Participants:**

This cohort study used data from the community-representative, prospective, longitudinal Great Smoky Mountains Study. Children and their parents were interviewed up to 8 times from January 1993 to December 2000 (ages 9-16 years; 6674 observations of 1420 participants) using the Child and Adolescent Psychiatric Assessment, which assessed parental incarceration, childhood psychiatric diagnoses, and other adversities. Young adults were followed up at ages 19, 21, 25, and 30 years from January 1999 to December 2015 (4556 observations of 1334 participants) to assess psychiatric diagnoses and functional outcomes indicative of a disrupted transition to adulthood. Data analysis was conducted from June 2018 to June 2019.

**Results:**

By age 16 years, 475 participants (weighted percentage, 23.9%) had a parental figure who had been incarcerated, including 259 young men (22.2%) and 216 young women (25.5%). Parental incarceration was associated with higher prevalence of childhood psychiatric diagnoses (eg, any depressive diagnosis: adjusted odds ratio [aOR], 2.5; 95% CI, 1.3-4.6; *P* = .006; attention-deficit/hyperactivity disorder: aOR, 2.3; 95% CI, 1.0-5.5; *P* = .06; and conduct disorder: aOR, 2.5; 95% CI, 1.4-4.3; *P* = .001). After accounting for childhood psychiatric diagnoses and adversity exposure, parental incarceration remained associated with increased odds of having an adult anxiety disorder (aOR, 1.7; 95% CI, 1.0-3.0; *P* = .04), having an illicit drug use disorder (aOR, 6.6; 95% CI, 2.6-17.0; *P* < .001), having a felony charge (aOR, 3.4; 95% CI, 1.8-6.5; *P* < .001), incarceration (aOR, 2.8; 95% CI, 1.4-5.4; *P* = .003), not completing high school (aOR, 4.4; 95% CI, 2.2-8.8; *P* < .001), early parenthood (aOR, 1.7; 95% CI, 1.0-3.0; *P* = .04), and being socially isolated (aOR, 2.2; 95% CI, 1.2-4.0; *P* = .009).

**Conclusions and Relevance:**

This study suggests that parental incarceration is associated with a broad range of psychiatric, legal, financial, and social outcomes during young adulthood. Parental incarceration is a common experience that may perpetuate disadvantage from generation to generation.

## Key Points

**Question:**

Is parental incarceration associated with increased odds of offspring receiving psychiatric diagnoses during young adulthood and experiencing obstacles that can derail a successful transition to adulthood (eg, in health, legal, financial, and social domains)?

**Findings:**

This cohort study, using data from a community-representative, longitudinal study, found that parental incarceration was associated with young adults’ increased odds of having an anxiety disorder, having a felony charge, spending time in jail, not completing high school, becoming a parent when younger than 18 years, and being socially isolated.

**Meaning:**

The findings suggest that parental incarceration is associated with offspring’s functional outcomes during young adulthood.

## Teaser

This cohort study examines whether parental incarceration is associated with increased levels of psychiatric diagnosis and poor outcomes among young adults.

## Introduction

During the last 50 years, US incarceration rates have grown tremendously. Recent point prevalence estimates suggest that 8% of US children younger than 18 years experienced the incarceration of a parent or guardian in 2016.^1^ Cumulatively, across their childhood years, many more children are exposed to the incarceration of a parental figure; children from racial and ethnic minority groups and disadvantaged backgrounds are disproportionally affected.^2,3^ Research suggests that the effects of specific childhood adversities can be transmitted across generations.^4^ This could also apply to parental incarceration, which could contribute to the intergenerational transmission of health, educational, and socioeconomic disparities.

During early and middle childhood, paternal incarceration is a risk factor for aggressive^5,6,7,8,9,10^ and antisocial^11^ behaviors but not for internalizing problems.^5,6,11,12^ From adolescence onward, evidence on the outcomes of parental incarceration is more limited, but existing studies suggest that parental incarceration is a risk factor for adolescents’ higher internalizing and externalizing problem scores,^7^ self-injurious behaviors, suicide attempts, and self-reported diagnosis and treatment of a mental, emotional, or behavioral problem.^13^ Likewise, in young adulthood, parental incarceration is associated with higher rates of depression^14,15,16^ and increased rates of anxiety and posttraumatic stress disorder among adult offspring.^14,15^

Although informative, prior studies typically have not accounted for childhood psychiatric status and adversities, which could underlie the association of parental incarceration with later mental and behavioral health problems.^17^ Furthermore, previous work typically assessed offspring mental health with questionnaires^7,13,16,18,19^ or respondent recall of diagnoses,^14,15^ which cannot generate *Diagnostic and Statistical Manual of Mental Disorders* (Fourth Edition) (*DSM*-*IV*) diagnoses and insight into clinical need. Rigorous studies are needed to clarify the independent association of parental incarceration with later health functioning.

Perhaps as salient for well-being as mental health is young adults’ ability to function in multiple areas of life (eg, health, avoidance of problems with the law, and economic and social well-being). Yet, emerging evidence from large national data sets suggests that parental incarceration is a risk factor for young adults’ forgoing health care, engaging in risky behaviors (eg, prescription drug misuse or many sexual partners),^20^ and worse health (eg*,* high cholesterol, asthma, HIV/AIDS, serious injuries).^14,15^ Likewise, experiencing parental incarceration heightens young adults’ risk of criminality,^21,22^ not completing high school^23^ or college,^15,24^ early parenthood,^25^ receiving welfare,^15^ and lower earnings or income.^15,26^ Commonly, these studies lack information on young adults’ previous psychiatric history, which could account for these associations.

The primary study question was whether parental incarceration was associated with increased levels of psychiatric diagnosis or poor outcomes (eg, in health, legal, financial, and social domains) in adulthood. Important nuances were examined, as follows: (1) the incarcerated parent’s biological relationship to the child, (2) for biological parents, whether the child resided with the incarcerated parent, (3) the incarcerated parent’s sex, (4) the child’s sex, and (5) the child’s race/ethnicity.

## Methods

### Participants

This report follows the Strengthening the Reporting of Observational Studies in Epidemiology (STROBE) reporting guideline for cohort studies.^27^ Data were drawn from the Great Smoky Mountains Study (GSMS), a longitudinal, community-representative cohort in which participants were followed up from age 9 years into adulthood. The GSMS, which enrolled children in 11 predominantly rural North Carolina counties, was originally designed to estimate the prevalence of mental illness and service use.^28^ Initially, 3 cohorts of children, aged 9, 11, and 13 years, were recruited from a pool of approximately 12 000 children using a 2-stage sampling design, resulting in 1420 participants (630 girls [weighted percentage, 49.0%]).^28^ Sampling weights were applied to adjust for differential probability of selection. An ascertainment figure appears in eFigure 1 in the Supplement, and the original study articles^28,29,30^ provide additional detail on sampling and derivation of sample weights.

Annual assessments were completed until participants were aged 16 years and again at ages 19, 21, 25, and 30 years for a total of 11 230 total assessments from January 1993 to December 2015. Across all assessments, 83.0% of possible interviews (11 230 of 13 530) were completed. Race/ethnicity was determined based on parent report. Before all interviews, parents and children signed institutional review board–approved informed consent and assent forms. All procedures and protocols for the present study were approved by the Duke University institutional review board.

### Childhood Variables

Childhood variables were aggregated into 1 observation per participant from all child and parent reports from ages 9 to 16 years and included the following events in childhood^1^: (1) parental incarceration status and subtype,^2^ (2) childhood psychiatric and substance use disorders, and (3) childhood adversities or hardships.^3^ All constructs were assessed using the structured Child and Adolescent Psychiatric Assessment (CAPA)^31,32^ and coded as present if reported by either the parent or child at any childhood observation.

### Parental Incarceration Status and Subtype

Participants and their primary caregivers were asked if any of the participants’ parental figures had ever been arrested as an adult (aged >18 years). If they answered affirmatively, they were asked to identify the worst result of the arrest (response options were not guilty, probation and/or community service, treatment order, fine, and prison or house arrest). Based on a review of interview notes, house arrest was so rarely reported that we assumed that those who reported prison or house arrest spent time incarcerated.^33^

Parental incarceration was considered affirmed if the participant or caregiver identified at least 1 parental figure as having ever spent time in prison, before or during the participant’s lifetime. Parental figures referred to any adult who had assumed responsibility for the child’s discipline or care, including parents (biological parents, stepparents, and adoptive parents) or other relatives. Parental figures also referred to adults who no longer lived with the child, which likely more accurately reflects modern family structures than assessments of residential or biological parents only.

Childhood psychiatric disorders and other adversities or hardships were assessed using the CAPA. This tool focuses on the 3 months preceding the interview to minimize recall bias. To create *DSM-IV* diagnoses, scoring programs combined information about the date of onset, duration, and symptom intensity. Psychiatric disorders assessed were anxiety disorders, mood disorders, conduct disorder, oppositional defiant disorder, attention-deficit/hyperactivity disorder, and substance use disorders. Test-retest reliability and validity of the CAPA diagnoses are similar to other psychiatric interviews.^31,32^

The co-occurrence of childhood adversities could confound the effects of parental incarceration during childhood on adult outcomes. Therefore, models controlled for childhood adversities, which were assessed at each observation. These included the following: (1) low socioeconomic status (including low educational attainment), (2) unstable family structure (eg, single parent family; divorce; or presence of stepparent), (3) family dysfunction (eg, inadequate parental supervision; domestic violence; high levels of parental conflict; maternal depression; marital relationship characterized by apathy, indifference, or high conflict; or high conflict between parent and child), (4) bullying, and (5) maltreatment (eg, physical abuse; sexual abuse; or neglect)^34^ (eAppendix in the Supplement).

### Adult Outcomes

All adult outcomes were coded as present if reported by a participant at any adult observation (aged 19, 21, 25, and 30 years), unless otherwise stated. All adult observations on a given outcome (ie, up to 4 observations per person) were aggregated into a single observation per participant.

Young adult psychiatric disorders were assessed using the Young Adult Psychiatric Assessment,^35^ an extension of the CAPA interview administered to participants aged 19 to 30 years. Assessments resembled those of childhood disorders, but used self-reports only. Disorders included any *DSM*-*IV *anxiety disorder and depressive disorder. Substance use disorders were coded for alcohol, cannabis, and other illegal drugs (eg, opioid or cocaine). For substance use disorders, we focused on ages 25 and 30 years, when substance use is not widespread but associated with a poor long-term prognosis.^36,37^

Young adult derailments assessed outcomes that typically impede functioning for an extended period of time and, therefore, could disrupt attaining important milestones during the transition to adulthood (Table 1). All derailments were assessed using the Young Adult Psychiatric Assessment^35^ and coded positive if reported during any adult assessment, with the exception of serious criminal activity, which was coded as present based on official criminal records between ages 16 and 25 years.

**Table 1.** Definitions and Prevalence of Adult Derailments

Derailment^a^	Prevalence, No. (%)^b^	Definition
**Health**		
Serious physical event	411 (27.2)	Participant reported diagnosis of serious physical illness or serious accident that involved risk of death or chronic disability
Psychiatric and substance use disorder	139 (9.3)	Participant met full criteria for both an emotional disorder and a substance abuse disorder
Suicidality	72 (7.0)	Participant reported recurrent thoughts of death (not just fear of dying), recurrent ideation, a suicide attempt, or specific plan for committing suicide
**Legal**		
Serious criminal activity	209 (17.7)	Official record of felony charge between ages 16 and 25 y
Incarceration	180 (8.2)	Participant reported time spent in jail or prison across adult assessments
**Financial**		
Did not complete high school	166 (9.7)	Participant had not received high school diploma, equivalent degree, or GED by last adult observation
Unable to keep job	70 (4.0)	Participant reported being fired from ≥3 jobs over the course of adult observations
Low income	272 (21.8)	Participant fell below poverty line at ages 25 or 30 y
**Social**		
Early parenthood	95 (5.5)	Participant reported becoming a parent prior to age of majority or legal adulthood, ie, age 18 y
No social support	234 (12.9)	Participant reported no best friend or confidante, little to no relationship with parents, and rare contact with peers across all adult observations
**Derailments**		
≥2	469 (26.0)	Participant reported ≥2 derailments
≥3	259 (14.0)	Participant reported ≥3 derailments

Abbreviation: GED, general equivalency diploma.

^a^ Except for serious criminal activity, derailments were assessed using the Young Adult Psychiatric Assessment.

^b^ Numbers are unweighted, and percentages are weighted.

### Statistical Analysis

All analyses applied sampling weights. Therefore, results represent unbiased estimates of the original population from which the sample was drawn. Consistent with common conventions, the results present weighted percentages and unweighted sample sizes. Weighted logistic (for binary outcomes such as psychiatric status) and Poisson (for count variables such as number of derailments) regression models were used to examine differences in adult outcomes by parental incarceration status for participants up to age 16 years. Statistical significance was set at *P* < .05, and all tests were 2-tailed. All models used SAS version 9.4 PROC GENMOD (SAS Institute) with robust variance (sandwich type) estimates derived from generalized estimating equations to account for the stratified sampling design.^38,39,40^

## Results

### Children’s Exposure to Parental Incarceration

Childhood parental incarceration reports were aggregated from up to 8 assessments of parents and children (ie, from up to 16 separate informant reports from child ages of 9-16 years). By age 16 years, 475 participants (23.9%) had a parental figure who had been incarcerated. Male and female children were similarly exposed to parental incarceration (259 [22.2%] vs 216 [25.5%]; *P* = .27), but these rates differed for white and nonwhite children. Based on parent report, 983 participants (89.4%) were white; 88 (6.9%) were African American, and 349 (3.7%) were American Indian. Specifically, 266 white children (21.4%) were exposed to parental incarceration compared with 42 African American children (42.7%) and 167 American Indian children (47.9%) (eFigure 2 in the Supplement).

At any single encounter, only 10.6% (95% CI, 8.7%-12.4%) of participants reported an incarcerated parent, which is similar to estimates derived from cross-sectional studies of parental incarceration. The overall parental incarceration estimate was also lower when parent or child reports only were used (19.9% for parent report; 15.1% for child report); agreement between informants was moderate to high (κ = 0.56).

In 139 parental incarceration cases (24.6%), multiple parents had been incarcerated, as GSMS defined parental figure as any adult who assumed responsibility for the child’s discipline or care. Overall, 18 African American participants (11.8%) and 48 American Indian participants (13.8%) had multiple incarcerated parents. Parental incarceration cases overwhelmingly involved male parents only (387 incarcerations [87.9%]), followed by male and female parents (55 [8.5%]), followed by female parents only (33 [3.5%]).

In most cases, the incarcerated parent was living outside of the child’s home (243 incarcerated parents [51.2%]). Almost one-third of incarcerated parents were residential parents, living in the home (147 [31.0%]), although parental incarceration could have occurred at any point after the parent was older than 18 years. In contrast, 85 parental childhood incarceration cases (17.9%) involved parents living both in and out of the home (eg, a nonresidential biological father and a residential stepfather). Finally, the incarcerated parent was typically a biological parent (338 [71.2%]). Most commonly, the incarcerated parent was a biological father outside of the home (183 incarcerations [51.3%]), followed by a biological father living in the home (85 [16.0%]), followed by a nonbiological father figure (typically, a stepparent) either in or out of the home. No other scenario accounted for more than 3% of parental figure incarcerations.

### Parental Incarceration and Offspring Childhood Psychiatric Disorders and Adversities or Hardships

After adjusting for sex and race/ethnicity, parental incarceration was associated with a higher likelihood of almost every childhood emotional and behavioral disorder except anxiety and substance disorders (any diagnosis: adjusted odds ratio [aOR], 2.3; 95% CI, 1.5-3.5; *P* < .001; any depressive diagnosis: aOR 2.5; 95% CI, 1.3-4.6; *P* = .006; attention-deficit/hyperactivity disorder: aOR, 2.3; 95% CI, 1.0-5.5; *P* = .06; oppositional defiant disorder: aOR, 2.7; 95% CI, 1.6-4.4; *P* < .001; conduct disorder: aOR, 2.5; 95% CI, 1.4-4.3; *P* = .001) (Table 2**)**. Parental incarceration was also associated with every type of childhood adversity or hardship except bullying (low family socioeconomic status: aOR, 4.2; 95% CI, 2.8-6.3; *P* < .001; family instability, aOR, 2.9; 95% CI, 1.9-4.4; *P* < .001; family dysfunction: aOR, 2.1; 95% CI, 1.4-3.2; *P* < .001; maltreatment: aOR, 3.3; 95% CI, 2.1-5.1; *P* < .001). There was little evidence that increased risk was sex-specific. The number of incarcerated parents was strongly associated with increased levels of almost all childhood psychiatric disorders and adversities or hardships (11 of 12 associations significant; eTable 1 in the Supplement), but there were fewer significant associations with biological status (5 of 12 associations significant; eTable 2 in the Supplement), sex of parent (2 of 12 associations significant; eTable 3 in the Supplement), or current residential status of the incarcerated parent (8 of 12 associations significant; eTable 4 in the Supplement).

**Table 2.** Prevalence and Association of Parental Incarceration With Childhood Diagnostic Groups and Adversities or Hardships

Diagnostic Group or Adversity	No. (%)^a^		OR (95% CI)^b^	*P* Value	*P* Value for Sex Difference^c^
	No Incarceration (n = 945)	Any Parental Figure Incarcerated (n = 475)			
**Psychiatric Problems**					
Any diagnosis	299 (23.0)	231 (39.9)	2.3 (1.5-3.5)	<.001	.45
Any anxiety diagnosis	108 (9.2)	86 (14.4)	1.7 (1.0-2.9)	.07	.79
Any depressive diagnosis	74 (6.1)	66 (13.0)	2.5 (1.3-4.6)	.006	.23
ADHD	52 (2.7)	26 (5.1)	2.3 (1.0-5.5)	.06	.14
ODD	123 (7.4)	115 (17.1)	2.7 (1.6-4.4)	<.001	.82
Conduct disorder	103 (6.6)	100 (14.7)	2.5 (1.4-4.3)	.001	.59
Substance disorder	54 (6.5)	54 (5.8)	0.9 (0.5-1.8)	.83	.50
**Adversities**					
Low family SES	335 (25.6)	314 (60.7)	4.2 (2.8-6.3)	<.001	.76
Family instability	241 (20.6)	229 (44.3)	2.9 (1.9-4.4)	<.001	.78
Family dysfunction	268 (23.3)	208 (38.4)	2.1 (1.4-3.2)	<.001	.44
Bullying	270 (24.3)	151 (31.9)	1.5 (1.0-2.3)	.07	.46
Maltreatment	158 (13.5)	164 (34.1)	3.3 (2.1-5.1)	<.001	.41

Abbreviations: ADHD, attention-deficit/hyperactivity disorder; ODD, oppositional defiant disorder; OR, odds ratio; SES, socioeconomic status.

^a^ Numbers are unweighted, and percentages are weighted.

^b^ Adjusted for sex and race/ethnicity.

^c^ Sex differences were assessed with an interaction term between sex and parental incarceration status.

### Parental Incarceration and Offspring Young Adult Outcomes

In total, 1334 participants (93.9%) were interviewed at least once in adulthood at ages 19, 21, 25, or 30 years. Participation rates in adulthood did not differ by childhood parental incarceration status. After adjusting for sex and race/ethnicity, parental incarceration was associated with offspring adulthood anxiety (aOR 2.4; 95% CI, 1.4-4.0; *P* = .001) and substance use disorders (aOR, 2.0; 95% CI, 1.2-3.3; *P* = .009) but not with depressive disorders (aOR, 1.4; 95% CI, 0.7-2.6; *P* = .31) (Table 3). In adjusted models, parental incarceration was still associated with anxiety and with all types of substance use disorders, especially those involving illicit drugs (aOR, 5.3; 95% CI, 2.3-12.2; *P* < .001). After adjusting for childhood psychiatric disorders and exposure to adversity, parental incarceration remained associated with increased odds of having an adult anxiety disorder (aOR, 1.7; 95% CI, 1.0-3.0; *P* = .04) and an illicit drug use disorder (aOR, 6.6; 95% CI, 2.6-17.0; *P* < .001).

**Table 3.** Prevalence and Association of Childhood Parental Incarceration With Offspring Adult Psychiatric Diagnoses

Diagnosis	No. (%)^a^		OR (95% CI)^b^	*P* Value	OR (95% CI)^c^	*P* Value	*P* Value for Sex Difference^d^
	No Incarceration (n = 893)	Any Parental Figure Incarcerated (n = 441)					
Any anxiety diagnosis	138 (13.8)	79 (27.6)	2.4 (1.4-4.0)	.001	1.7 (1.0-3.0)	.04	.52
Any depressive diagnosis	108 (9.3)	49 (13.3)	1.4 (0.7-2.6)	.31	0.9 (0.5-1.6)	.63	.80
Substance use disorder diagnosis	142 (18.5)	106 (29.5)	2.0 (1.2-3.3)	.009	2.5 (1.4-4.3)	.002	.53
Alcohol use disorder diagnosis	72 (11.7)	47 (13.9)	1.3 (0.7-2.6)	.42	2.2 (1.0-4.8)	.05	.74
Cannabis use disorder diagnosis	55 (5.7)	49 (14.4)	2.1 (0.8-5.0)	.12	3.5 (1.7-7.3)	<.001	.12
Illicit drug use disorder diagnosis	33 (2.7)	46 (11.4)	5.3 (2.3-12.2)	<.001	6.6 (2.6-17.0)	<.001	.04

Abbreviation: OR, odds ratio.

^a^ Numbers are unweighted, and percentages are weighted.

^b^ Adjusted for sex and race/ethnicity.

^c^ Adjusted for sex, race/ethnicity, childhood psychiatric disorders, and childhood adversities. Child psychiatric disorders include anxiety, depression, attention-deficit/hyperactivity disorder, conduct disorder, oppositional defiant disorder, and substance use disorders. Childhood adversities include low family socioeconomic status, familial instability, family dysfunction, maltreatment, and bullying.

^d^ Sex differences were assessed with an interaction term between sex and parental incarceration status.

Next, we examined associations of parental incarceration with health, legal, financial, and social outcomes that can derail a successful transition to adulthood (Table 4). Parental incarceration was not associated with health outcomes, but it was associated with increased legal (felony charge: aOR, 4.9; 95% CI, 2.7-8.8; *P* < .001; incarceration: aOR, 4.5; 95% CI, 2.4-8.4; *P* < .001), financial and educational (not completing high school: aOR, 7.0; 95% CI, 3.8-12.7; *P* < .001; experiencing financial strain: aOR, 2.7; 95% CI, 1.6-4.4; *P* = .001), and social outcomes (early parenthood: aOR, 5.6; 95% CI, 2.6-12.3; *P* < .001; social isolation: aOR, 2.9; 95% CI, 1.7-5.0; *P* = .001). Except for financial strain, these associations persisted after accounting for childhood psychiatric disorders and family adversities (ie, low socioeconomic status, family instability, family dysfunction, bullying, and maltreatment); parental incarceration was associated with increased odds of having a felony charge (aOR, 3.4; 95% CI, 1.8-6.5; *P* < .001), not completing high school (aOR, 4.4; 95% CI, 2.2-8.8; *P* < .001), early parenthood (aOR, 1.7; 95% CI, 1.0-3.0; *P* = .04), and being socially isolated (aOR, 2.2; 95% CI, 1.2-4.0; *P* = .009). Compared with children who did not experience parental incarceration, children with an incarcerated parent were more likely to report multiple (ie, ≥2 and ≥3) derailments while transitioning to adulthood (≥2 derailments: aOR, 1.9; 95% CI, 1.2-3.1; *P* = .01; ≥3 derailments: aOR, 3.1; 95% CI, 1.8-5.5; *P* < .001). The number of significant associations of parental incarceration with sex were few.

**Table 4.** Prevalence and Association of Childhood Parental Incarceration and Offspring Adult Derailments

Derailment	No. (%)^a^		OR (95% CI)^b^	*P* Value	OR (95% CI)^c^	*P* Value	*P* Value for Sex Difference^d^
	No Incarceration (n = 893)	Any Parental Figure Incarcerated (n = 441)					
**Health**							
Serious health problem	246 (25.6)	165 (32.5)	1.4 (0.9-2.1)	.13	1.1 (0.7-1.7)	.57	.83
Psychiatric and substance problem	55 (5.5)	42 (9.6)	2.0 (1.0-3.9)	.05	1.6 (0.8-3.0)	.18	.68
Suicidal behavior	49 (7.0)	23 (7.1)	1.0 (0.4-2.4)	.97	0.7 (0.2-2.1)	.52	.43
**Legal**							
Felony charge	96 (11.5)	113 (35.0)	4.9 (2.7-8.8)	<.001	3.4 (1.8-6.5)	<.001	.13
Incarceration	76 (5.1)	104 (18.7)	4.5 (2.4-8.4)	<.001	2.8 (1.4-5.4)	.003	.82
**Financial**							
Did not complete high school	73 (5.0)	93 (25.0)	7.0 (3.8-12.7)	<.001	4.4 (2.2-8.8)	<.001	.41
Unable to keep job	41 (3.4)	29 (6.0)	1.6 (0.6-3.9)	.34	1.3 (0.5-3.6)	.62	.10
Low income	153 (17.5)	119 (37.2)	2.7 (1.6-4.4)	.001	1.6 (0.9-2.8)	.10	.95
**Social**							
Early parenthood	43 (2.8)	52 (14.3)	5.6 (2.6-12.3)	<.001	3.0 (1.1-8.4)	.04	.05
No social support	131 (9.4)	103 (24.5)	2.9 (1.7-5.0)	.001	2.2 (1.2-4.0)	.009	.97
**Derailments**							
≥2	235 (20.4)	224 (44.1)	2.9 (1.9-4.4)	<.001	1.9 (1.2-3.1)	.01	.88
≥3	109 (5.5)	141 (31.5)	4.8 (2.9-7.9)	<.001	3.1 (1.8-5.5)	<.001	.56

Abbreviation: OR, odds ratio.

^a^ Numbers are unweighted, and percentages are weighted.

^b^ Adjusted for sex and race/ethnicity.

^c^ Adjusted for sex, race/ethnicity, childhood psychiatric disorders, and childhood adversities. Child psychiatric disorders include anxiety, depression, attention-deficit/hyperactivity disorder, conduct disorder, oppositional defiant disorder, and substance use disorders. Childhood adversities include low family socioeconomic status, familial instability, family dysfunction, maltreatment, and bullying.

^d^ Sex differences were assessed with an interaction term between sex and parental incarceration status.

Biological relatedness to the incarcerated parent was not associated with increased risk for psychiatric disorders or derailments (Table 5). Having a higher number of incarcerated parents was a risk factor for adult psychopathology and more derailments (eTable 5 in the Supplement); eTable 6 in the Supplement shows the associations of having a mother incarcerated and having a father incarcerated compared with no parental incarceration. In no case was the observed association significantly different between maternal and paternal incarceration. Finally, living with a previously incarcerated parent was associated with increased risk of having a substance disorder in adulthood, but this increased risk did not extend to emotional disorders or derailments (eTable 7 in the Supplement).

**Table 5.** Prevalence and Association of Biological Status of Incarcerated Parent and Adult Diagnoses and Derailments

Diagnosis or Derailment	No. (%)^a^			OR (95% CI), Biological Parent vs No Incarceration	*P* Value	OR (95% CI), Nonbiological Parent vs No Incarceration	*P* Value	*P* Value for Difference^b^
	No Incarceration (n = 893)	Biological Parent (n = 311)	Nonbiological Parent (n = 65)					
**Psychiatric problems**								
Any anxiety diagnosis	138 (13.8)	57 (26.9)	9 (27.6)	1.9 (1.0-3.3)	.04	1.5 (0.6-4.0)	.43	.63
Any depressive diagnosis	108 (9.3)	33 (11.1)	9 (21.5)	0.7 (0.4-1.4)	.34	1.8 (0.6-5.1)	.27	.33
Any substance use disorder diagnosis	142 (18.5)	73 (29.4)	33 (26.7)	2.4 (1.3-4.4)	.006	3.3 (1.2-9.2)	.02	.55
**Derailments**								
≥2	235 (24.2)	150 (39.9)	74 (58.8)	1.7 (1.0-2.8)	.05	4.1 (1.4-11.7)	.008	.07
≥3	109 (8.5)	195 (32.7)	46 (43.5)	3.0 (1.7-5.3)	<.001	6.0 (1.9-18.5)	.002	.35

Abbreviation: OR, odds ratio.

^a^ Numbers are unweighted, and percentages are weighted. Participants with both an incarcerated biological parent and nonbiological parent were excluded from this analysis.

^b^ The difference column tested the difference between the biological and nonbiological groups.

### Follow-up Analyses

It is plausible that the effects of parental incarceration varied as a function of combination between sex of the child and the incarcerated parent. With few mother-only cases, we could only test whether the associations of paternal incarceration were stronger for boys or girls. There was no evidence that paternal incarceration was associated with increased risk for boys or girls selectively in terms of psychiatric or substance disorders, overall derailments, or specific derailments. Finally, we tested whether observed associations were affected by informant effects (ie, whether parent or child had reported the parental incarceration); eTable 8 in the Supplement shows that associations reported above were significant (and of similar magnitude) regardless of whether the parent or the child reported the parental incarceration.

## Discussion

The incarceration of a parent represents a serious disruption of a child’s life. Our results revealed that the incarceration of a parent figure was common, disproportionally so in African American and American Indian families, a risk factor for anxiety and substance use disorders a decade or more later, and associated with significant hurdles during the transition to adulthood, including having a felony charge, spending time incarcerated, not completing high school, becoming a parent when younger than 18 years, and being socially isolated. These associations remained when accounting for a broad range of other childhood psychiatric status and adversities such as poverty and maltreatment, suggesting that childhood parental incarceration has an enduring reach into offspring’s adult lives.

Notably, the GSMS prevalence of parental incarceration was higher than in other population-based samples (eg, 8% of parents in the National Survey of Children’s Health^1^; 10.7% of biological parents in the National Longitudinal Study of Adolescent to Young Adult Health^20^). However, this is not surprising. The GSMS used a prospective longitudinal design with up to 8 assessments of parental incarceration from 2 reporters (ie, up to 16 data points, spread over several years to calculate parental incarceration status per child). Such designs typically result in more accurate assessments of risk exposures compared with designs using single-point retrospective assessments that are prone to recall bias and forgetting.^41^ Indeed, when GSMS parental incarceration rates were constrained to lifetime estimates at single assessments, they averaged 10.6%, which was similar to the rates reported from nationally representative samples.

Our study documented the long reach of childhood parental incarceration. With respect to psychiatric disorders, offspring’s adult rates remained elevated more than a decade later. Risk of substance use disorders was also elevated—a novel finding considering that past studies had mostly focused on prescription drug^20^ and illicit drug use^26^ but not at the diagnostic level. Interestingly, living with a previously incarcerated parent posed a greater risk for young adult substance use disorders compared with the incarceration of a nonresidential parent. It is possible that previously incarcerated parents who lived in the same home socialized offspring into substance use patterns and/or provided access.^42^

Our findings regarding derailments painted a concerning picture of intergenerational transmission of criminal involvement and incarceration. However, this is not necessarily biologically based; indeed, it was found for both biological and nonbiological incarcerated parents.^21,22,26^ Consistent with previous work,^25^ our study also suggests that children of incarcerated parents spend less time in school, with potential adverse consequences for lifetime career opportunities and earnings. Daughters of incarcerated parents were at heightened risk for becoming young mothers—a finding broadly consistent with previous research^25^ reporting that paternal incarceration (which 87.9% of children with incarcerated parents experienced in our study) was associated with having a child by age 23 years, although that study did not differentiate this finding by child’s sex. Interestingly, our study also revealed that children of incarcerated parents were at increased risk of lack of social support and connection in their young adult years. This is concerning considering social connection in adulthood is crucial for well-being and health and could also contribute to opportunities in other life domains such as finding a stable job.^43^

### Limitations

This study has limitations. Although community representative, GSMS is not representative of the US population. The data lack exact incarceration dates and duration and do not distinguish between incarcerations that occurred in jails or state and federal prisons. The developmental timing of parental incarceration was not considered. Parents’ residential status reflected the time of the interview, but incarceration could have occurred in the past. Further, with all observational studies, it is important that findings be retested in independent samples.^44^

## Conclusions

Parental incarceration appears to cast a long shadow on offspring’s adult years. These associations may be partly explained by the traumatic separation from a parent or loved one, the stigma of having a parent incarcerated, the short- and long-term economic ramifications that may occur from parental incarceration, or other factors. Our findings are informative about the potentially high societal costs of incarcerating children’s caregivers—potentially for generations to come. From a public health perspective, preventing exposure to parental incarceration could improve the well-being of children and young adults, as could aiding children and families affected by the incarceration of a parental figure.

## Notes

Supplement. **eAppendix. **Definition of Childhood Covariates **eTable 1. **Prevalence and Associations Between Number of Incarcerated Parents and Childhood Diagnoses and Other Adversities **eTable 2**. Prevalence and Associations Between Biological Status of Incarcerated Parent and Childhood Diagnoses and Other Adversities **eTable 3. **Prevalence and Associations Between Sex of Incarcerated Parent and Childhood Diagnoses and Other Adversities **eTable 4. **Prevalence and Associations Between In/Out-of-Home Status of Incarcerated Parent and Childhood Diagnoses and Other Adversities **eTable 5. **Prevalence and Associations Between Number of Incarcerated Parents and Adult Diagnoses and Derailments **eTable 6. **Prevalence and Associations Between Sex of Incarcerated Parent and Adult Diagnoses and Derailments **eTable 7. **Prevalence and Associations Between In/Out-of-Home Status of Incarcerated Parent and Adult Diagnoses and Derailments **eTable 8. **Associations Between Child and Parent Report Parental Incarceration Status and Adult Diagnoses and Derailments **eFigure 1. **Ascertainment of the Original Great Smoky Mountains Study Sample **eFigure 2. **Parental Incarceration Subtype by Child’s Race/Ethnicity
