# Supplementary material for: Association of Parental Incarceration With Psychiatric and Functional Outcomes of Young Adults
Source: JAMA Netw Open. 2019 Aug 23;2(8):e1910005. doi: 10.1001/jamanetworkopen.2019.10005 (PMC6714027; doi:10.1001/jamanetworkopen.2019.10005)
Supplement: Supplement. — eAppendix. Definition of Childhood Covariates eTable 1. Prevalence and Associations Between Number of Incarcerated Parents and Childhood Diagnoses and Other Adversities eTable 2. Prevalence and Associations Between Biological Status of Incarcerated Parent and Childhood Diagnoses and Other Adversities eTable 3. Prevalence and Associations Between Sex of Incarcerated Parent and Childhood Diagnoses and Other Adversities eTable 4. Prevalence and Associations Between In/Out-of-Home Status of Incarcerated Parent and Childhood Diagnoses and Other Adversities eTable 5. Prevalence and Associations Between Number of Incarcerated Parents and Adult Diagnoses and Derailments eTable 6. Prevalence and Associations Between Sex of Incarcerated Parent and Adult Diagnoses and Derailments eTable 7. Prevalence and Associations Between In/Out-of-Home Status of Incarcerated Parent and Adult Diagnoses and Derailments eTable 8. Associations Between Child and Parent Report Parental Incarceration Status and Adult Diagnoses and Derailments eFigure 1. Ascertainment of the Original Great Smoky Mountains Study Sample eFigure 2. Parental Incarceration Subtype by Child’s Race/Ethnicity [file jamanetwopen-2-e1910005-s001.pdf]

Supplementary Online Content

Gifford EJ, Eldred Kozecke L, Golonka M, et al. Association of parental incarceration with psychiatric and functional outcomes of young adults. *JAMA Netw Open*. 2019;2(8):e1910005. doi:10.1001/jamanetworkopen.2019.10005

eAppendix. Definition of Childhood Covariates

eTable 1. Prevalence and Associations Between Number of Incarcerated Parents and Childhood Diagnoses and Other Adversities

eTable 2. Prevalence and Associations Between Biological Status of Incarcerated Parent and Childhood Diagnoses and Other Adversities

eTable 3. Prevalence and Associations Between Sex of Incarcerated Parent and Childhood Diagnoses and Other Adversities

eTable 4. Prevalence and Associations Between In/Out-of-Home Status of Incarcerated Parent and Childhood Diagnoses and Other Adversities

eTable 5. Prevalence and Associations Between Number of Incarcerated Parents and Adult Diagnoses and Derailments

eTable 6. Prevalence and Associations Between Sex of Incarcerated Parent and Adult Diagnoses and Derailments

eTable 7. Prevalence and Associations Between In/Out-of-Home Status of Incarcerated Parent and Adult Diagnoses and Derailments

eTable 8. Associations Between Child and Parent Report Parental Incarceration Status and Adult Diagnoses and Derailments

eFigure 1. Ascertainment of the Original Great Smoky Mountains Study Sample

eFigure 2. Parental Incarceration Subtype by Child's Race/Ethnicity

This supplementary material has been provided by the authors to give readers additional information about their work.

© 2019 Gifford EJ et al. *JAMA Network Open.*

eAppendix. Definition of Childhood Covariates

Five types of family adversities/hardships were assessed: (1) low socioeconomic status (SES), (2) family instability, (3) family dysfunction, (4) peer victimization and (5) maltreatment. Low SES was positive if the child's family met 2 or more of the following conditions: below the US federal poverty line based upon household size and income, highest level of parental education was high school, or low parental occupational prestige (40). Family instability was positive if child's family met 2 or more of the following conditions: single parent structure, step-parent in household, divorce, parental separation, or change in parent structure. Family dysfunction was positive if child's family met 5 or more of the following conditions: inadequate parental supervision of child's free time, over-involvement of the parent into the child's activities in an age-inappropriate manner, physical violence between parents, top 20% in terms of frequency of parental arguments, marital relationship characterized by absence of affection, apathy, or indifference, child is upset by or actively involved in arguments between parents, mother scores in elevated range on depression questionnaire, top 20% in terms of frequency of arguments between parent and child, and most parental activities are source of tension or worry for the child. Peer victimization was positive if child or parent reported that the child had been a regular target of bullying or harassment by peers. Maltreatment was positive if child or parent reported that the child had been physically abused (subject victim of intentional physical violence by family member), sexually abused (subject involved in activities for purposes of perpetrators sexual gratification including kissing, fondling, oral-genital, oral-anal, genital or anal intercourse), or neglected by parents (caregiver unable to meet child's need for food, clothing, housing, transportation, medical attention or safety). Codebooks for all items available at http://devepi.duhs.duke.edu/codebooks.html.

© 2019 Gifford EJ et al. *JAMA Network Open.*

eTable 1. Prevalence and Associations Between Number of Incarcerated Parents and Childhood Diagnoses and Other Adversities.

	No incarceration	1 parent	2 + parents	Adjusted for sex, race		
**Overall**	n=945	n=336	n=139	OR	CI	p
**Psychiatric problems**	% (n)	% (n)	% (n)			
Any diagnosis	23.0 (299)	37.2 (147)	48.6 (84)	**1.9**	**1.4-2.5**	**<0.001**
Any anxiety diagnosis	9.2 (108)	11.3 (51)	24.6 (35)	**1.6**	**1.2-2.3**	**0.004**
Any depressive diagnosis	6.1 (74)	14.0 (43)	9.8 (23)	**1.7**	**1.2-2.3**	**0.002**
ADHD	2.7 (52)	5.8 (19)	2.9 (7)	**1.6**	**1.0-2.4**	**0.04**
ODD	7.4 (123)	16.3 (81)	19.7 (34)	**1.9**	**1.4-2.5**	**<0.001**
Conduct Disorder	6.6 (103)	13.3 (59)	19.2 (41)	**2.0**	**1.5-2.7**	**<0.001**
Substance Disorder	6.5 (54)	4.3 (30)	10.4 (24)	1.2	0.8-1.8	0.38
**Adversities**						
Low family SES	25.6 (335)	54.7 (208)	80.3 (106)	**3.1**	**2.3-4.2**	**<0.001**
Family instability	20.6 (241)	37.2 (133)	67.1 (96)	**2.4**	**1.8-3.2**	**<0.001**
Family dysfunction	23.3 (268)	33.7 (137)	53.5 (71)	**1.9**	**1.4-2.4**	**<0.001**
Peer victimization	24.3 (270)	29.7 (102)	38.9 (49)	**1.4**	**1.1-1.8**	**0.02**
Maltreatment	13.5 (158)	28.4 (102)	52.5 (62)	**2.5**	**1.9-3.3**	**<0.001**

All percentages are weighted and ns are unweighted. Bolded values indicate statistically significant difference at p <0.05. ADHD = attention-deficit/hyperactivity disorder; ODD = oppositional defiant disorder.

© 2019 Gifford EJ et al. *JAMA Network Open.*

eTable 2. Prevalence and Associations Between Biological Status of Incarcerated Parent and Childhood Diagnoses and Other Adversities.

	No incarceration	Bio parent	Nonbio parent	Bio parent vs. no incarceration			Nonbio parent vs. no incarceration			Diff?
**Overall**	n=945	n=338	n=67							
**Psychiatric problems**	% (n)	% (n)	% (n)	OR	CI	p	OR	CI	p	p
Any diagnosis	23.0 (299)	37.9 (31)	38.4 (31)	**2.1**	**1.3-3.3**	**0.002**	2.2	1.0-5.2	0.07	0.89
Any anxiety diagnosis	9.2 (108)	13.6 (58)	8.5 (11)	1.6	0.8-3.0	0.16	0.9	0.4-2.2	0.81	0.22
Any depressive diagnosis	6.1 (74)	12.8 (45)	15.4 (10)	**2.5**	**1.2-4.9**	**0.01**	2.8	0.7-11.0	0.15	0.82
ADHD	2.7 (52)	3.8 (16)	11.6 (4)	1.6	0.6-4.4	0.37	**6.5**	**1.4-30.5**	**0.02**	0.09
ODD	7.4 (123)	14.4 (75)	28.3 (19)	**2.1**	**1.2-3.8**	**0.01**	**5.3**	**2.0-14.0**	**<0.001**	0.10
Conduct Disorder	6.6 (103)	14.3 (67)	9.9 (11)	**2.3**	**1.2-4.3**	**0.01**	1.8	0.8-4.0	0.16	0.61
Substance Disorder	6.5 (54)	5.1 (34)	5.1 (8)	0.8	0.4-1.8	0.61	0.8	0.3-2.1	0.65	0.96
**Adversities**										
Low family SES	25.6 (335)	58.3 (218)	59.3 (44)	**3.7**	**2.4-5.9**	**<0.001**	**4.3**	**1.6-11.3**	**<0.001**	0.68
Family instability	20.6 (241)	37.2 (140)	64.9 (41)	**2.2**	**1.3-3.5**	**0.001**	**7.0**	**2.7-18.0**	**<0.001**	**0.02**
Family dysfunction	23.3 (268)	35.7 (138)	44.2 (30)	**1.9**	**1.2-3.0**	**0.007**	**2.6**	**1.1-6.6**	**0.04**	0.49
Peer victimization	24.3 (270)	27.1 (99)	38.6 (25)	1.1	0.7-1.9	0.54	2.1	0.8-5.2	0.11	0.28
Maltreatment	13.5 (158)	31.6 (108)	24.1 (19)	**3.0**	**1.8-4.8**	**<0.001**	2.0	0.7-5.7	0.19	0.54

All percentages are weighted and ns are unweighted. Bolded values indicate statistically significant difference at $p < 0.05$. Participants with both an incarcerated biological parent and nonbiological parent were excluded from this analyses. The difference column tested the difference between the biological and nonbiological groups. ADHD = attention-deficit/hyperactivity disorder; ODD = oppositional defiant disorder.

© 2019 Gifford EJ et al. *JAMA Network Open.*

eTable 3. Prevalence and Associations Between Sex of Incarcerated Parent and Childhood Diagnoses and Other Adversities

	No incarceration	Father	Mother	Father vs. no incarceration			Mother vs. no incarceration			Diff?
**Overall**	n=945	n=387	n=33	OR	CI	p	OR	CI	p	
**Psychiatric problems**	% (n)	% (n)	% (n)							
Any diagnosis	23.0 (299)	38.5 (180)	27.0 (12)	**2.2**	**1.4-3.3**	**<0.001**	1.3	0.5-3.4	0.62	0.30
Any anxiety diagnosis	9.2 (108)	13.1 (68)	7.3 (4)	1.5	0.8-2.7	0.18	0.8	0.2-3.1	0.74	0.41
Any depressive diagnosis	6.1 (74)	13.4 (54)	9.9 (4)	**2.5**	**1.3-4.9**	**0.007**	1.9	0.5-7.9	0.39	0.69
ADHD	2.7 (52)	5.3 (22)	0.0 (0)	2.4	1.0-5.9	0.06	**--**	**--**	**--**	**--**
ODD	7.4 (123)	16.5 (91)	14.8 (6)	**2.5**	**1.5-4.4**	**<0.001**	2.3	0.7-7.1	0.16	0.84
Conduct Disorder	6.6 (103)	14.3 (78)	1.2 (1)	**2.4**	**1.3-4.3**	**0.004**	0.2	0.0-1.3	0.09	**0.01**
Substance Disorder	6.5 (54)	5.2 (39)	3.7 (3)	0.8	0.4-1.7	0.63	0.6	0.2-2.1	0.39	0.34
**Adversities**										
Low family SES	25.6 (335)	58.3 (243)	95.0 (33)	**3.8**	**2.5-5.9**	**<0.001**	**47.4**	**12.6-178.2**	**<0.001**	**<0.001**
Family instability	20.6 (241)	41.7 (169)	70.3 (21)	**2.6**	**1.7-4.1**	**<0.001**	**8.2**	**2.8-24.3**	**<0.001**	**0.03**
Family dysfunction	23.3 (268)	36.9 (168)	23.4 (11)	**2.0**	**1.3-3.1**	**0.002**	1.1	0.4-3.2	0.89	0.25
Peer victimization	24.3 (270)	30.3 (121)	45.9 (7)	1.4	0.9-2.2	0.16	3.0	0.8-11.4	0.12	0.23
Maltreatment	13.5 (158)	32.1 (126)	29.1 (10)	**3.0**	**1.9-4.8**	**<0.001**	2.7	0.9-8.5	0.08	0.83

All percentages are weighted and ns are unweighted. Bolded values indicate statistically significant difference at p <0.05. Participants with both an incarcerated mother and father figure were excluded from this analyses. The difference column tested the difference between the mother and father figure groups. ADHD = attention-deficit/hyperactivity disorder; ODD = oppositional defiant disorder.

© 2019 Gifford EJ et al. *JAMA Network Open.*

eTable 4. Prevalence and Associations Between In/Out-of-Home Status of Incarcerated Parent and Childhood Diagnoses and Other Adversities

	No incarceration	In home	Out of home	In home vs. no incarceration			Out of home vs. no incarceration			Diff?
**Overall**	n=945	n=147	n=243	OR	CI	p	OR	CI	p	p
**Psychiatric problems**	% (n)	% (n)	% (n)							
Any diagnosis	23.0 (299)	38.7 (71)	35.8 (108	**2.1**	**1.1-3.9**	**0.03**	**2.0**	**1.2-3.3**	**0.009**	0.89
Any anxiety diagnosis	9.2 (108)	12.7 (23)	10.5 (40)	1.5	0.6-4.0	0.40	1.2	0.6-2.3	0.68	0.71
Any depressive diagnosis	6.1 (74)	11.5 (18)	13.2 (31)	2.1	0.7-5.9	0.18	**2.5**	**1.2-5.4**	**0.02**	0.63
ADHD	2.7 (52)	13.0 (10)	2.8 (14)	**5.3**	**1.6-17.6**	**0.006**	1.2	0.6-2.6	0.62	**0.03**
ODD	7.4 (123)	17.5 (35)	15.8 (56)	**2.6**	**1.2-5.5**	**0.02**	**2.5**	**1.3-4.6**	**0.006**	0.96
Conduct Disorder	6.6 (103)	8.7 (24)	14.8 (48)	1.2	0.6-2.4	0.56	**2.5**	**1.3-5.1**	**0.009**	0.06
Substance Disorder	6.5 (54)	4.3 (16)	4.8 (23)	0.6	0.3-1.3	0.18	0.8	0.3-2.0	0.65	0.40
**Adversities**										
Low family SES	25.6 (335)	60.9 (103)	58.9 (147)	**4.3**	**2.0-8.9**	**0.001**	**3.9**	**2.4-6.4**	**<0.001**	0.66
Family instability	20.6 (241)	33.6 (58)	43.6 (110)	1.9	0.9-3.9	0.08	**2.8**	**1.7-4.7**	**<0.001**	0.35
Family dysfunction	23.3 (268)	37.2 (72)	35.9 (96)	**2.0**	**1.0-3.9**	**0.05**	**2.0**	**1.2-3.2**	**0.01**	0.90
Peer victimization	24.3 (270)	30.0 (45)	32.3 (80)	1.4	0.7-2.8	0.38	1.5	0.9-2.5	0.13	0.81
Maltreatment	13.5 (158)	21.7 (41)	36.0 (89)	1.9	0.9-3.9	0.09	**3.5**	**2.1-5.9**	**<0.001**	0.20

All percentages are weighted and ns are unweighted. Bolded values indicate statistically significant difference at p <0.05. Participants with both an incarcerated parent in and out of the home were excluded from this analyses. The difference column tested the difference between the in and out of the home groups. ADHD = attention-deficit/hyperactivity disorder; ODD = oppositional defiant disorder.

© 2019 Gifford EJ et al. *JAMA Network Open.*

**eTable 5. Prevalence and Associations Between Number of Incarcerated Parents and Adult Diagnoses and Derailments**						
	No incarceration	1 parent	2 + parents	Adjusted for sex, race, psych. dx. and adversities		
	% (n)	% (n)	% (n)	OR	CI	p
**Total**	76.9 (893)	17.4 (308)	5.8 (133)			
**Psychiatric problems**						
Any anxiety dx.	13.8 (138)	27.6 (54)	27.3 (25)	1.4	0.9-2.0	0.12
Any depressive dx.	9.3 (108)	10.9 (33)	20.5 (16)	1.0	0.6-1.6	0.99
Any substance dx.	18.5 (142)	31.8 (73)	21.3 (33)	**1.7**	**1.2-2.4**	**0.004**
**Derailments**						
2+	20.4 (235)	20.3 (150)	56.4 (74)	**1.6**	**1.1-2.2**	**0.005**
3+	5.5 (109)	29.0 (195)	39.7 (46)	**1.9**	**1.4-2.8**	**<0.001**

All percentages are weighted and ns are unweighted. Child psychiatric disorders includes depression, anxiety, ADHD, conduct, oppositional defiant and substance disorders. Childhood adversities/hardships include low SES, familial instability, family dysfunction, maltreatment and peer victimization. Bolded values are significant at $p < 0.05$.

© 2019 Gifford EJ et al. *JAMA Network Open.*

eTable 6. Prevalence and Associations Between Sex of Incarcerated Parent and Adult Diagnoses and Derailments										
	No incarceration	Father	Mother	Father vs. no incarceration			Mother vs. no incarceration			Diff?
	% (n)	% (n)	% (n)	OR	CI	p	OR	CI	p	p
**Total**	76.9 (893)	20.2 (358)	0.8 (30)							
**Psychiatric problems**										
Any anxiety dx.	13.8 (138)	27.2 (61)	48.8 (8)	1.8	1.0-3.1	0.04	**5.1**	**1.3-19.9**	**0.02**	0.21
Any depressive dx.	9.3 (108)	12.8 (39)	13.4 (4)	0.9	0.5-1.6	0.62	1.0	0.2-4.7	0.98	0.79
Any substance dx.	18.5 (142)	31.2 (88)	18.5 (7)	**2.6**	**1.4-4.6**	**0.002**	1.7	0.5-6.1	0.39	0.20
**Derailments**										
2+	24.2 (235)	41.8 (175)	63.2 (17)	**1.8**	**1.1-3.0**	**0.03**	**3.8**	**1.2-12.7**	**0.03**	0.42
3+	8.5 (109)	30.7 (111)	52.2 (12)	**3.1**	**1.8-5.6**	**<0.001**	**6.7**	**1.8-24.8**	**0.004**	0.40

All percentages are weighted and ns are unweighted. Participants with both an incarcerated mother and father figure were excluded from this analyses. The difference column tested the difference between the mother and father figure groups. Bolded values are significant at p <0.05.

© 2019 Gifford EJ et al. *JAMA Network Open.*

**eTable 7. Prevalence and Associations Between In/Out-of-Home Status of Incarcerated Parent and Adult Diagnoses and Derailments**										
	No incarceration	In home	Out of home	In home vs. no incarceration			Out of home vs. no incarceration			Diff?
	% (n)	% (n)	% (n)	OR	CI	p	OR	CI	p	p
**Total**	76.9 (893)	20.2 (358)	0.8 (30)							
**Psychiatric problems**										
Any anxiety dx.	13.8 (138)	25.4 (24)	29.1 (39)	1.7	0.8-3.6	0.17	**1.9**	**1.0-3.6**	**0.04**	0.85
Any depressive dx.	9.3 (108)	13.6 (10)	13.9 (31)	1.1	0.4-3.2	0.82	0.9	0.5-1.7	0.79	0.66
Any substance dx.	18.5 (142)	39.7 (35)	26.5 (47)	**3.4**	**1.4-8.5**	**0.009**	**2.1**	**1.1-4.0**	**0.02**	**0.03**
**Derailments**										
2+	24.2 (235)	45.3 (68)	43.5 (115)	**2.3**	**1.0-5.3**	**0.05**	**1.9**	**1.1-3.3**	**0.03**	0.48
3+	8.5 (109)	32.1 (43)	31.6 (73)	**3.7**	**1.5-9.0**	**0.004**	**3.1**	**1.7-5.9**	**<0.001**	0.58

All percentages are weighted and ns are unweighted. Participants with an incarcerated parent both in and out of the home were excluded from this analyses. The difference column tested the difference between the in and out of the home groups. Bolded values are significant at $p < 0.05$.

© 2019 Gifford EJ et al. *JAMA Network Open.*

eTable 8. Associations Between Child and Parent Report Parental Incarceration Status and Adult Diagnoses and Derailments

	Parent-report			Child-Report		
	OR	CI	p	OR	CI	p
**Total**						
**Psychiatric problems**						
Any anxiety dx.	**1.9**	**1.1-3.4**	**0.02**	**1.8**	**1.0-3.1**	**0.04**
Any depressive dx.	**0.8**	**0.4-1.5**	**0.42**	**0.8**	**0.4-1.5**	**0.50**
Any substance dx.	**2.7**	**1.5-4.8**	**0.001**	**2.3**	**1.3-4.3**	**0.006**
Derailments						
2+	**1.7**	**1.0-2.8**	**0.04**	**1.9**	**1.1-3.1**	**0.02**
3+	**2.5**	**1.4-4.3**	**0.002**	**2.8**	**1.6-5.0**	**<0.001**

All percentages are weighted and ns are unweighted. Bolded values are significant at $p < 0.05$.

© 2019 Gifford EJ et al. *JAMA Network Open.*

eFigure 1. Ascertainment of the Original Great Smoky Mountains Study Sample

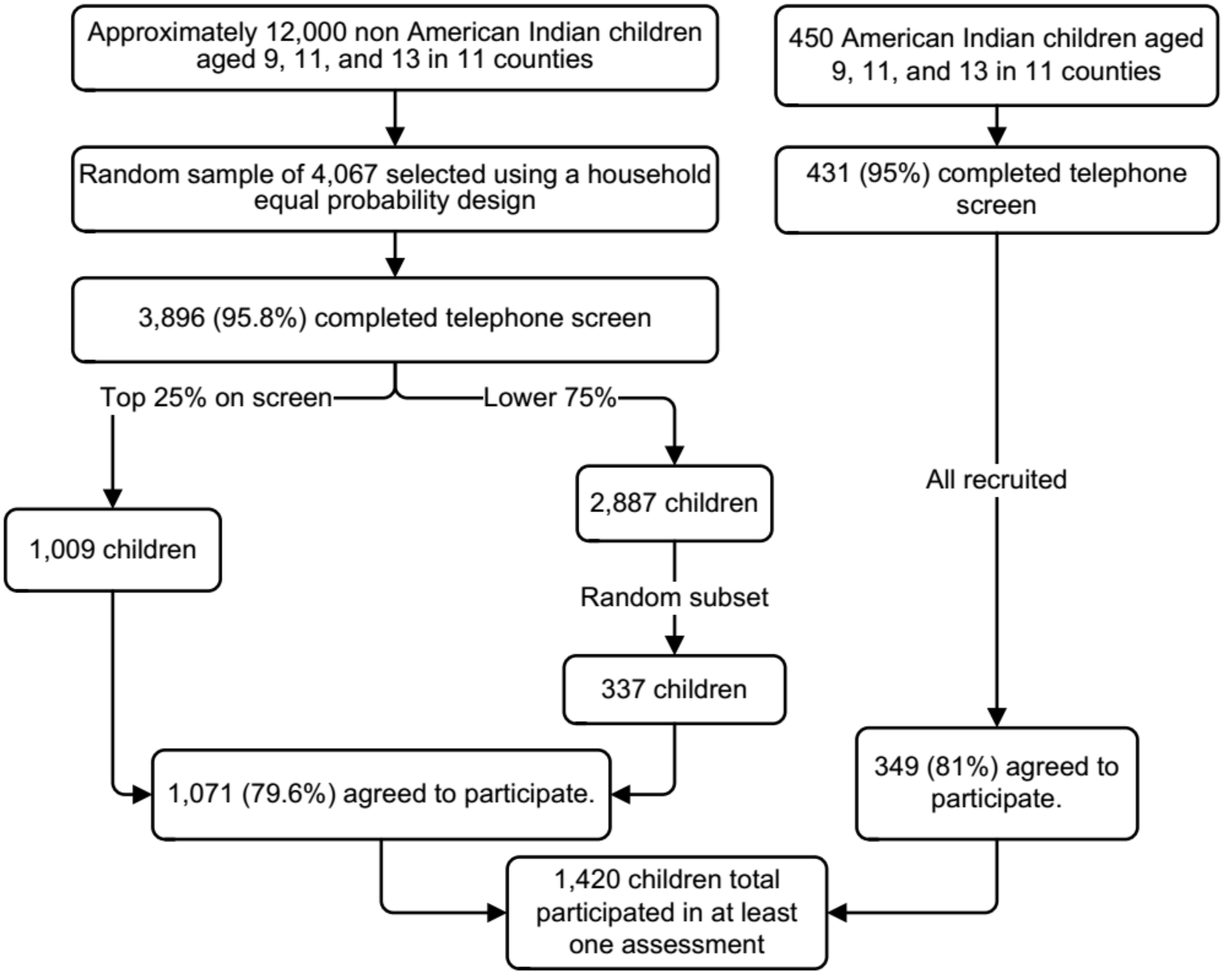

© 2019 Gifford EJ et al. *JAMA Network Open.*

eFigure 2. Parental Incarceration Subtype by Child's Race/Ethnicity

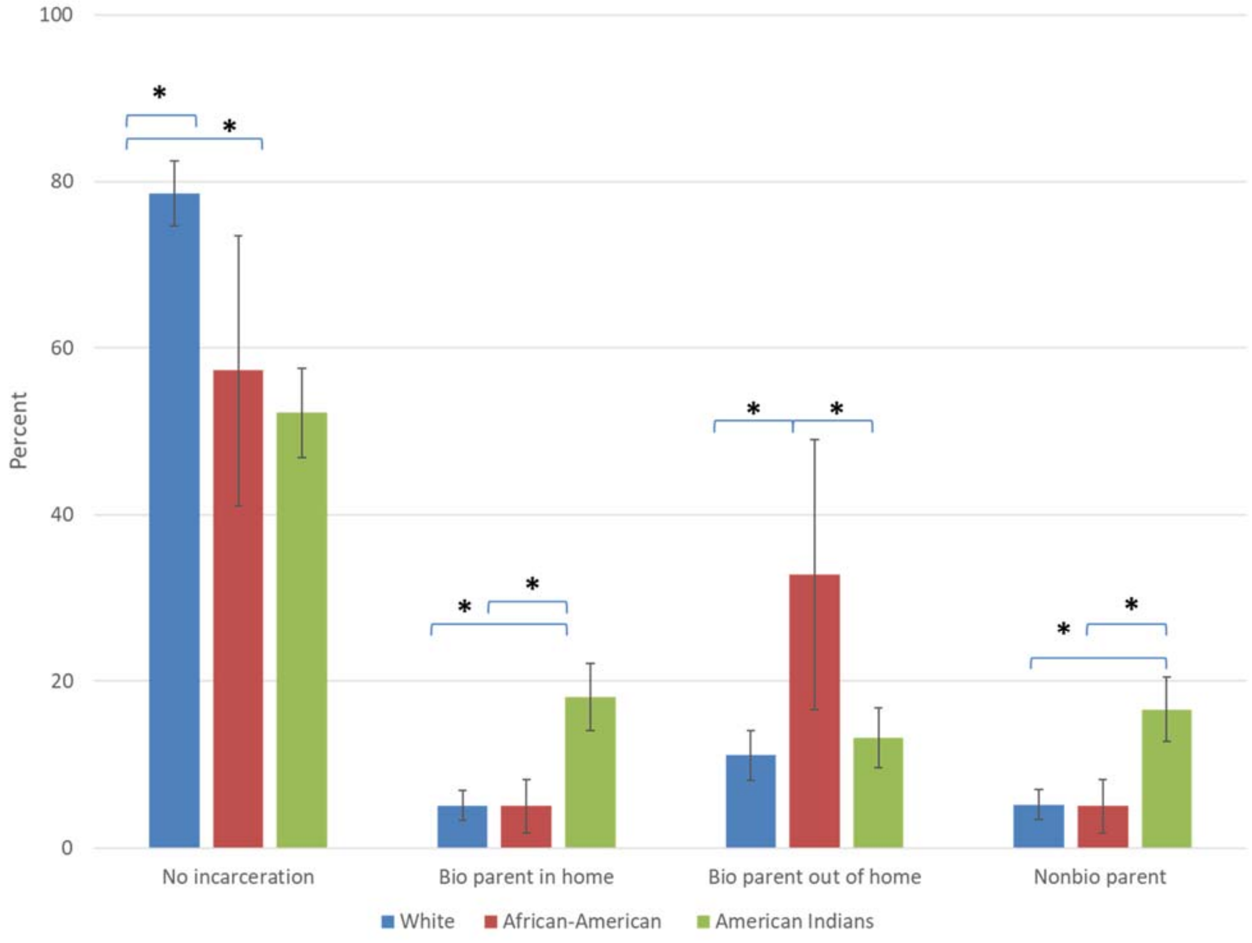

© 2019 Gifford EJ et al. *JAMA Network Open.*
